# Linking planetary boundaries and ecosystem accounting, with an illustration for the Colombian Orinoco river basin

**DOI:** 10.1007/s10113-018-1282-1

**Published:** 2018-02-12

**Authors:** Leonardo Vargas, Louise Willemen, Lars Hein

**Affiliations:** 1 Environmental Systems Analysis Group, Wageningen University and Research, PO Box 47, 6700 AA Wageningen, The Netherlands; 2 Faculty of Geo-Information Science and Earth Observation (ITC), University of Twente, PO Box 217, 7500 AE Enschede, The Netherlands

**Keywords:** Ecosystem accounting, Planetary boundaries, Natural resource management, Ecosystem services

## Abstract

Economic development has increased pressures on natural resources during the last decades. The concept of planetary boundaries has been developed to propose limits on human activities based on earth processes and ecological thresholds. However, this concept was not developed to downscale planetary boundaries to sub-global level. The absence of boundaries at sub-global levels constrains the use of the concept in environmental governance and natural resource management, because decisions are typically taken at these levels. Decisions at the national level are currently supported, among others, by statistical frameworks in particular the System of National Accounts. However, statistical frameworks were not developed to compile environmental information, hindering environmental decision making. Our study examines if and how ecosystem accounting can be used in combination with the concept of planetary boundaries in guiding human activities at the level of a river basin. We assess the applicability of both frameworks for natural resource management in the Orinoco river basin, based on adaptive management components. Our analysis indicates that differences in the purpose of analysis, information provided, and methods constrain the potential integration of both frameworks. Nevertheless, the way both frameworks conceptualize the social system and the interactions between social and ecological systems can facilitate translating planetary boundaries into indicators considered in ecosystem accounting. The information recorded in national ecosystem accounts can support establishing ecological thresholds and, more fundamentally, to relate ecological thresholds to human impacts on ecosystem condition. Capitalizing on these synergies requires further exchange of experiences between the communities working on ecosystem accounting and planetary boundaries.

## Introduction

Economic growth has progressively increased human pressures on the earth system (Foley et al. 2005; Folke 2010; Steffen et al. 2011). Human pressures on the earth system have led to, among others, the modification of nitrogen, phosphorus, and water cycles, and changes in land cover and ecosystems (Carpenter 2005; Foley et al. 2005; MA 2003). The discussion on how to best reconcile economic development with sustainable natural resource management is still ongoing, but is reinforced by the increasing pressure on relatively undisturbed ecosystems in developing countries. Sustainable development is challenged by the complexity of the environmental problems derived from human and nature interactions. Complex environmental problems such as climate change and ocean acidification cannot be fully understood by separate disciplinary approaches; they demand integrative, multidisciplinary approaches (Liu et al. 2015; Ostrom 2009). Integrated approaches view human and nature as connected entities embedded in socio-ecological systems, interacting at multiple organizational (e.g., administrative arrangements), spatial (e.g., river basin), and temporal scales (e.g., years) (Berkes et al. 2008; Liu et al. 2007; Ostrom 2009). Liu et al. (2015) highlighted the development of quantitative frameworks as a significant contribution to better understand complex environmental problems in socio-ecological systems by assessing the connections between the socio-economic and the environmental components of the system. Integrated approaches such as used in the Intergovernmental Panel on Climate Change assessments and the Millennium Ecosystem Assessment incorporate quantitative frameworks to explore the links between global environmental and social-economic changes (MA 2003). However, few approaches incorporate quantitative frameworks to propose limits on economic activities within earth system functioning to reconcile economic development with sustainable natural resource management. Two complimentary integrated approaches aiming to reconcile economic development with sustainable natural resource management through quantitative frameworks are the planetary boundaries framework and ecosystem accounting.

The planetary boundaries framework identifies nine priority earth system processes: the nitrogen, phosphorus, carbon and water cycles, climate, stratosphere, land and ocean systems, biodiversity, aerosol loading, and chemical pollution (Rockström et al. 2009). The framework presents, for each of these earth system processes, quantified boundary levels that are associated to ecological thresholds. Crossing these thresholds would generate unacceptable environmental change (Rockström et al. 2009). The framework distinguishes between boundaries associated to continental or global thresholds, such as stratospheric ozone depletion, and boundaries based on processes with no evidence of planetary threshold behavior, such as water use. Boundaries associated to earth system processes with no evidence of planetary behavior are not quantified in the framework. However, because earth system comprises smaller-scale, spatially connected, interacting systems, crossing ecological thresholds in these small-scale systems can propagate and cause a shift in the whole system (Barnosky et al. 2012; Peters et al. 2009). The increasing awareness on irreversible changes in the earth functioning triggered by crossing ecological thresholds increase the influence of planetary boundaries in international discourses of global environmental governance and global sustainable development (e.g., United Nations 2030 Sustainable Development Agenda) (Griggs et al. 2013). However, the implications of using planetary boundaries in global environmental governance are challenged by uncertainties in the boundaries associated to unknown ecological thresholds arising at sub-global level, and by multilevel governance (Galaz et al. 2012). Decisions concerning environmental governance and natural resources management are mostly taken at the national and sub-national level, requiring multilevel governance between institutions, policies, and social organizations (Galaz et al. 2012; Häyhä et al. 2016; Nilsson and Persson 2012).

Ecosystem accounting has been developed under auspices of the United Nations Statistical Commission, synthetized in the System of Environmental Economic Accounting Experimental Ecosystem Accounting (SEEA-EEA) (United Nations et al. 2014b). Ecosystem accounting complements the international statistical standard for environmental economic accounting, the System of Environmental Economic accounting-2012 Central Framework (SEEA-Central Framework) (United Nations et al. 2014a). Ecosystem accounting organizes spatially explicit biophysical and monetary data in a set of tables and accounts in which different aspects of ecosystems and flows of ecosystem services are quantified and linked to economic activities. Information compiled in tables and accounts can be reported at the national and sub-national level, following the same concepts, definitions, and accounting rules synthetized in the SEEA-CF and the System of National Accounts (United Nations et al. 2014b). Recent studies demonstrate the potential use of ecosystem accounting information to support decision and policy making on land and resource management (e.g., water purification in Europe and monetary valuation of ecosystem services in The Netherlands (La Notte et al. 2017; Remme et al. 2015)).

The planetary boundaries and ecosystem accounting frameworks have a merit in supporting decision and policy making in natural resource management to achieve a more sustainable development; however, both frameworks have their own limitations. The planetary boundaries framework was not designed to be applied at the national and sub-national levels, hindering the ability to influence decision making at these levels. Ecosystem accounting can record a wide range of data sources (e.g., remote sensing and statistical information) at the national and sub-national level including ecological thresholds as indicators; however, there have as yet not been any ecosystem accounts that include such thresholds. Hence, there is a need to explore if both frameworks can be reconciled and to assess if experiences from both frameworks can be used to mutually reinforce one another.

The aim of this paper is to examine if and how planetary boundaries can be used in combination with ecosystem accounting in proposing limits on human activities at the level of a large river basin. In particular, this paper will compare and contrast the planetary boundaries and ecosystem accounting frameworks and illustrate if looking for similarities and differences provides complementary information for sustainable natural resource management in the Colombian Orinoco river basin. The Orinoco river basin is selected because of rapid ecosystem change ongoing in this area. Human activities such as oil palm and energy generation have been growing over the last two decades thereby increasing the pressure on large areas of relatively undisturbed tropical forests. The area is also subject to increasing withdrawal of water resources for irrigation and hydropower and the eutrophication of lakes and rivers.

## Method

### Comparing frameworks

We compare the planetary boundaries and ecosystem accounting frameworks using two sets of criteria based on Binder et al. (2013). First, we provide a general overview and discuss the frameworks based on contextual criteria, and second, we provide an in-depth comparison based on structural criteria. Contextual criteria provide information about the origin of the framework, the purpose, information provided, use in policy making, and spatial and temporal domains (Table 1). Structural criteria describe how the social and ecological systems are conceptualized, how they interact, and their analytical depth. The conceptualization of the social system includes the analysis of different hierarchical levels (e.g., individual, groups), how these levels interact, and how these levels are integrated. The conceptualization of the ecological system includes a (i) description on how the system is overviewed from an anthropocentric or eco-centric perspective and (ii) how the ecological dynamics are described (e.g., using natural language or specific equations). The conceptualization of the interaction between both systems is described using three forms of interaction: (i) if the ecological system influences the social system, (ii) if the social system influences the ecological system, and (iii) if reciprocity between both systems is considered (Binder et al. 2013) (Table 1). And lastly, we classify the frameworks as either analysis-oriented or action-oriented. Whereas the goal in analysis-oriented frameworks is to provide a general language to formulate and approach different research questions, the goal in action-oriented frameworks is to act or intervene.

**Table 1:** Criteria used to compare the Planetary Boundaries (PB) and Ecosystem Accounting (EA) frameworks

Contextual criteria	Question
Disciplinary origin	What discipline provides the starting point of the framework?
Theoretical origin	Which theories are the foundations of the framework? What is the motivation for applying these theories?
Purpose of analysis	What is the aim of the framework? What type of information is provided? How is the framework used in policy making?
Temporal domain	At what temporal level is the framework applied (years, months, days)?
Spatial domain	At what spatial level is the framework applied (global, national, sub-national)?
Structural criteria	Question
Social system	
• Name	How is the social system described in the framework?
• Level	What institutional level is included in the framework (individual, group, organizational, society)?
• Conceptualization and dynamics	How is the social system conceptualized? How are the interactions between the levels incorporated? Macro: depicts the social system only at macro level Micro: depicts the social system only at micro level Macro → micro: the macro level influence the micro level Micro → macro: focuses in the micro level and how this level impacts the macro level Macro↔ micro: duality between macro and micro levels
Ecological system	
• Name	How is the ecological system included in the framework?
• Level	At which spatial level is the framework designed (global, national, sub-national)?
• Conceptualization and dynamics	How is the ecological system conceptualized?
Social-ecological system	
• Conceptualization of interactions	How are the interactions between the levels of the ecological system incorporated? How are the dynamics and interactions between social and ecological systems conceptualized? E → S: ecological system influence the social system S → E: social system influence the ecological system E ↔ S: reciprocity between systems How are they measured?
• Depth of social and ecological system	Are the social and ecological systems treated as equal?
Orientation	Is the framework “analysis” or “action” oriented?
Adapted from Binder et al. (2013)	

### Assessing the applicability of both frameworks for natural resource management in the Colombian Orinoco river basin

#### The Orinoco basin

The Orinoco is a transboundary river basin between Colombia and Venezuela. The Colombian side of the river basin embraces 345,000 km^2^, collecting waters from the Andes mountains, the Guyana shield, and the Amazon river basin (Barletta et al. 2015; León 2005). Water resources include six rivers with an annual average discharge higher than 1,000 m^3^/s, 55% of the national wetlands, 33% of the national freshwater reserves, 40% underground waters, and 40% fish species (Correa et al. 2005; Romero-Ruiz et al. 2012). The river basin is an important reserve of tropical forests covering more than 80,000 km^2^ of different types of forest (Ideam 2011a). Fast changes are occurring in the river basin including deforestation, the introduction of exotic crops (e.g., oil palm, rice, soy), and the increase of improved grass species to raise cattle (Benavides 2010; Ideam 2011a; Sanchez-Cuervo and Aide 2013).

#### Adaptive management in the Orinoco river basin

Natural resource management in socio-ecological systems is challenging because the dynamic underlying social and ecological systems with potential non-linear feedbacks are difficult to predict and control (Armitage et al. 2015; Folke et al. 2002; Levin et al. 2013). Adaptive management of natural resources reduces the uncertainty in the impacts of different policy choices by making decisions in an iterative way over time (Holling 1978; Rist et al. 2013b; Walters 1986). We use adaptive management components described by Rist et al. (2013a) as criteria to compare the applicability of the planetary boundaries and ecosystem accounting frameworks for natural resource management in the Orinoco river basin (Table 2).

**Table 2:** Adaptive management criteria

Components	Question	Description
Stakeholders participation	Is the information useful to define stakeholders? How are stakeholders defined in the framework?	In this criterion, we report if the information provided by each framework can be used to define the people, organizations, and institutions who use, influence, and have an interest in the use of natural resources
Definition of the management problem	Is the information useful to define management objectives? How are management objectives defined and measured in the framework?	In this criterion, we assess if the information provided by each framework can be used to define clear management objectives, how it can be measured, and if this information can be used to assess the impacts of management actions
Establishment of a baseline of understanding	What type of information is supplied? How is the information collected and used? How are alternative management options defined?	In this criterion, we identify if the models used by each framework can provide useful information to evaluate alternative actions by stakeholders
Implementation of actions/policies	How can actions/policies be implemented?	In this criterion, we assess which actions/policies are defined from a set of possible alternatives, guided by management objectives adjusted according to possible changes
Monitoring effect	What monitoring strategies can be supported? What institutional arrangements can be defined for monitoring progress?	In this criterion, we assess the monitoring potential by evaluating if the information provided by each of the frameworks can be used to design an efficient monitoring system
Adapted from Rist et al. (2013a, b)		

##### Planetary boundaries and earth system functioning processes at sub-global level

To assess the applicability of planetary boundaries for adaptive management of natural resources in the river basin, we use three earth system processes with sub-global dynamics critical for earth system functioning—*biogeochemical flows of nitrogen (N) and phosphorus(P), land system change, and freshwater use*—(Steffen et al. 2015) (Table 3). These three processes are directly relevant for river basin management in the Orinoco.

**Table 3:** Earth system functioning processes of the Planetary Boundaries (PB) approach that operate at sub-global level

Earth functioning processes	Relevance of the earth system functioning process and main human pressures	Reference
Biogeochemical flows of nitrogen and phosphorous	Assimilable forms of nitrogen and phosphorous are currently included in the industrial production of synthetic fertilizer to increase the production of food, fibers, and biofuels. The turnover rate of nitrogen have doubled, and the annual application of phosphorus to agricultural ecosystems is about a third of which naturally cycle through all terrestrial ecosystems	(Carpenter 2005; Gruber and Galloway 2008; Steffen et al. 2015)
Land system change	Tropical forests play a significant role in global biophysical climate regulation by modulating the exchanges of energy and water between land surface and atmosphere. Deforestation in regional tropical forests influences global climate regulation by altering evapotranspiration patterns	(Chapin et al. 2008; Foley et al. 2003; Steffen et al. 2015)
Freshwater use	All terrestrial biomes depend on fresh water provided through land precipitation as part of the water cycle. Land precipitation returns water to the atmosphere via evapotranspiration (green water) without entering the terrestrial water cycle (blue water including stream flow and groundwater recharge and outflow). Human manipulation of the global water cycle affects ecosystem functioning, biodiversity, food, and human health, about 25% of the planetary river basins run dry before reaching the ocean because human water use (blue water)	(Bogardi et al. 2013; Molden et al. 2007; Steffen et al. 2015; Trenberth et al. 2007)

##### Ecosystem accounting

To assess the applicability of ecosystem accounting for adaptive management of natural resources in the river basin, we assess the information required to compile extent, condition, capacity, and ecosystem services that supply accounts following the structure and guidelines of the SEEA-EEA (Table 4) (United Nations et al. 2014b). We provide an illustration for the river basin, assessing two ecosystems (oil palm plantations and tropical forest) in terms of extent, condition, capacity to supply ecosystem services, and the supply of ecosystem services, based on the methods described in Hein et al. (2015) and Vargas et al. (2017) to obtain the different values for the different accounts.

**Table 4:** Information compiled in the different ecosystem accounting accounts

Ecosystem accounting accounts	Explanation
Extent	Defines ecosystem’s size and location, typically delineated be on land cover type
Condition	Reflects key ecosystem’s characteristics (e.g., hydrological and nutrient cycles, species composition, and productivity) that influence ecosystem’s extent, functioning, and quality
Ecosystem capacity to supply ecosystem services	Defines ecosystem’s ability to supply ecosystem services under current conditions without degrading the ecosystem. Ecosystem capacity to supply ecosystem services depends on ecosystem extent and condition, current and future ecosystem use patterns, and involves resource harvesting and regeneration
Ecosystem services supply	Reflects the supply of ecosystem services such as food, fibers, medicines, and fresh water from the different types of ecosystems. Ecosystem services are ecosystem’s contributions to benefits used in economic and other economic activity

Information contained in the different accounts is based on Hein et al. (2015) and United Nations et al. (2014a, b)

## Results

### Comparing frameworks

#### Description based on contextual criteria

##### Discipline origins

The origins of the planetary boundaries can be found in ecological economics, earth system science, and the literature on global change and on modeling complex ecological dynamics and ecological thresholds. Earth system science enables the identification of earth system functioning processes essential to maintain planet stability, and global change brings human activities as drivers of pressures in earth system. Ecological thresholds lead to abrupt irreversible transitions if they are crossed. They are crucial in setting limits on human activities. The complexity of the dynamics of ecological thresholds are summarized by splitting ecological thresholds in two categories, planetary thresholds driven by global processes and sub-global thresholds that arise at regional and local level. The impacts of crossing planetary thresholds are palpable at sub-global level, e.g., coastal areas are vulnerable for a rise in the sea level as a consequence of melting polar ice. The impacts of crossing ecological thresholds that arise at sub-global level aggregate, increasing the risk of crossing thresholds in other earth system processes. For example, the use of synthetic fertilizer containing nitrogen and phosphorus in farming areas increases the risk of eutrophication in downstream water resources, gradually increasing the risk of large-scale anoxia in the oceans, with potential consequences for biodiversity and other earth system functions (Watson et al. 2017). Rockström et al. (2009) recognized the spatial heterogeneity of many earth system processes, especially in those where sub-global dynamics play an important role, such as in the nitrogen, phosphorus, and water cycles. Of the various disciplinary bases of the planetary boundaries, economics is least visible. Economic information is not used in defining the boundaries, only to indicate economic development as a driver of the increasing pressures on earth system processes.

Ecosystem accounting is based on measurement concepts from different disciplines including statistics, ecology, spatial modeling, economics, and accounting. Ecology brings in concepts such as the ecosystem, ecosystem services, ecosystem processes, and biodiversity. Ecosystems are viewed as functional units from which plant, animals, and microorganisms interact with the non-living environment, generating goods and services for people (United Nations et al. 2014b). Ecosystem services are defined as the ecosystems’ contributions to human activities. Concepts such as resilience, complex dynamics, and ecological thresholds are included in the ecosystem accounting framework; however, the practical use of these concepts is not clearly indicated. Economic concepts such as production, consumption, accumulation, and ownership of assets are brought from economics. Economic concepts related to ecosystem assets and flows of ecosystem services underpin the accounting perspective of ecosystem accounting, enabling the establishment of trade-offs between generation and use of ecosystem services, and ecosystem’s potential to supply services in the future. The weak economic background perceived in the planetary boundaries framework can be strengthened by the strong economic background of ecosystem accounting. Likewise, earth system functioning, global change, and ecological thresholds are strongly embedded in the planetary boundaries, providing key information to be compiled in ecosystem accounting condition accounts.

##### Theoretical background

The development of the planetary boundaries framework was fueled by the new paradigm that states we have now entered a new era of global change known as the “Anthropocene,” driven by the rapid increase in human activities (Crutzen 2006; Rockström et al. 2009). The planetary boundaries theoretical background stems from earlier approaches to set limits on human activities including limits to growth, safe minimum standards, and the tolerable windows approach (Crowards 1998; Meadows et al. 1972; Raffensperger 1999). These approaches attempt to analyze and quantify natural boundaries using future scenarios and the application of the precautionary principle to avoid critical transitions. The theory behind the planetary boundaries differs from these approaches by focusing on earth system processes, the incorporation of associated ecological thresholds irrespective of human preferences, values and compromises, and the identification of boundaries from which humanity can take actions towards sustainable development. The development of ecosystem accounting was motivated by the fact that separate analysis of ecosystems and the economy do not fully encompass the relationship between human activities and the environment (United Nations et al. 2014b). A main premise is that individual and societal decisions concerning the use of environmental resources will be better informed by using integrated information connecting ecosystems to economic activities. Differences in the theoretical background between both frameworks can be seen as complementary. Whereas the planetary boundaries incorporate earth system processes in the human responsibility of defining limits for social and economic growth, ecosystem accounting provides environment and economic spatially explicit integrated information to inform human society on how to improve the use of natural resources.

##### Purpose of analysis

The purpose of analysis in the planetary boundaries framework is to propose a safe space in which human activities can take place while avoiding the transgression of critical ecological thresholds. The framework is implemented through expert assessments and synthesis of scientific knowledge. The framework provides information to set boundary levels through control variables (e.g., km^3^ of water use per year). Although the planetary boundaries concept is not meant for targeting a specific institution, different international scientific-policy initiatives have used the concept (e.g., the Global Environmental Outlook 5) (Galaz et al. 2012). The purpose of analysis in ecosystem accounting is to integrate environmental and economic information to support policy making and environmental management. Information is presented in physical and monetary terms, and organized following the same logic used in standard measurements of the economy (e.g., national accounts). From a measurement perspective, ecosystem accounting focuses on the (i) flows of ecosystem services and (ii) changes on the stock of ecosystems, i.e., *ecosystem assets*. Global initiatives such as the World Bank Wealth Accounting and the Valuation of Ecosystem Services (WAVES) and The Economics of Ecosystems and Biodiversity (TEEB) are users of the framework. Ecosystem accounting can provide broader measures of progress and sustainable development and can be used for policy in public areas of concern such as land and resource management (United Nations et al. 2014b). Although both frameworks pursue different purposes, supporting the achievement of sustainable development can be seen as a common ground between the two frameworks. Whereas the planetary boundaries aim to influence current trends in social and economic development, bringing a new concept to achieve global sustainability, ecosystem accounting supports national economic decision and policy making to achieve a sustainable use of natural resources.

#### Description based on structural criteria

##### Social system

The social system in the planetary boundaries framework is conceptualized from an anthropocentric perspective. The institutional level addressed in the framework is the global society, referenced as “humanity” and “global community.” The hierarchical level at which the social system is conceptualized is the macro level using a global perspective, disregarding interactions with lower hierarchical levels such as groups, organizations, and individual persons. Humanity is perceived as a dominant force shaping the planet, and as the main driver of global change. Humanity determines the level and values of the planetary boundaries to maintain a safe distance from critical ecological thresholds. The distance is determined by normative judgements based on risk and uncertainty measures. Likewise, the social system in ecosystem accounting framework is conceptualized from an anthropocentric perspective. The institutional levels addressed in the framework are the institutional units recording statistical information. These institutional units include establishments, enterprises, government entities, and households. Institutional units can be grouped in industries (economic units with similar economic activities) and sectors (economic units with similar purposes, objectives, and behaviors). The hierarchical level at which the social system is conceptualized is a duality between macro and micro levels. For example, changes in the economic behavior of consumption of goods and services at household level influence economic activity at the level of industry, and likewise, government decisions influence the industry and households. The accounting structure applied in ecosystem accounting to conceptualize the social system through institutional units can be used to support implementing planetary boundaries at lower hierarchical levels than “humanity,” including enterprises and government entities at the national level.

##### Ecological system

It can be argued that the ecological system is conceptualized from an eco-centric perspective in the planetary boundaries framework because the biophysical processes controlling the earth self-regulating capacity, and the ecological thresholds associated to these processes occur irrespective of human activities. Human activities are perceived as *embedded* in the earth system, and strongly depend on critical earth system processes (Heikkurinen et al. 2016). Ecological dynamics in the framework are described by control variables which are quantifiable units used to estimate a boundary level for each earth system process, and the safest distance from an ecological threshold. Expert assessments and biophysical data determine the value of the control variables. The ecological system is conceptualized from an anthropocentric perspective in ecosystem accounting, regarding the central role given to ecosystem services, defined as the contributions of ecosystems to benefits used in economic and other human activities (United Nations et al. 2014b). This definition has a profound anthropocentric perspective, as without human beneficiaries, the flow of ecosystem services will be zero. Human activities are perceived as embedded within ecosystems, recognizing that human activities influence ecosystems across the planet. The assessment of ecosystems includes key characteristics such as structure (e.g., food webs, habitats), composition (e.g., fauna, flora), processes (e.g., photosynthesis), and functions (e.g., nutrients recycling). Measurements in the ecological system include assessments of changes in the stock of ecosystem assets and flows between ecosystem assets in physical and monetary terms, recorded in ecosystem’s extent, condition, and capacity accounts.

##### Interactions between social and ecological systems

The interactions between the social and ecological systems are conceptualized by describing planetary boundaries for human activities that pressure key earth system functioning processes in the planetary boundaries framework. The dynamics of the interaction is reciprocal, human activities pressure earth system processes, and feedbacks from earth system affect human society. The framework is action-oriented because the main goal is to influence current development strategies by proposing limits on human activities. Conversely, the interactions between the social and ecological systems are conceptualized through the lens of ecosystem services in ecosystem accounting. The dynamics of the interaction is conceptualized as the ecosystem system influencing the social system by providing flows of ecosystem services that benefit human society. Although human activities modify ecosystems, often to influence the supply of ecosystem services (e.g., irrigation systems support crops during the dry season), ecosystems influence human society by regulating the stream of ecosystem services that benefit human society. Changes in the stock of ecosystem assets and flows between ecosystem assets conceptualize the ecological system; however, flows from ecosystem assets to economic and non-economic activities conceptualize the dynamics of the interaction between the social and ecological system. Measurements include flows from ecosystem assets to economic and non-economic activities, measured in physical and monetary terms and recorded in ecosystem services supply accounts.

### The applicability of both frameworks for adaptive natural resource management in the Orinoco river basin

This section summarizes the role of the planetary boundaries and ecosystem accounting frameworks for each adaptive management component presented in Table 2.

#### Stakeholder participation

The information provided by the planetary boundaries framework is not meant to identify stakeholders; however, the participation of stakeholders is central for implementing policy actions in natural resource management. For the Orinoco example, stakeholders are connected to economic activities that generate impacts on the nitrogen and phosphorus cycles, water use, and land system change. These economic activities include among others oil palm and rice production, cattle ranching, timber harvesting, and hydropower generation (Benavides 2010). Stakeholders involved in these activities include farmers, farmers associations (e.g., FEDEPALMA, FEDEGAN), government institutions (e.g., municipalities), and hydropower industry (e.g., Chivor S.A). Likewise, the spatially explicit biophysical data recorded in ecosystem accounting tables and accounts is not meant to directly identify stakeholders. However, the economic statistical information covering economic transactions between households, legal entities, and government institutions compiled in institutional units can be used to identify stakeholders that impact ecosystems (and are affected by changes in ecosystems) in the river basin. Legal entities include the production-oriented groups, such as farmers associations (e.g., FEDEARROZ, FEDEPALMA) and non-profit organizations. In the Orinoco basin, relevant government institutions include environmental corporations such as Corpochivor and Cormacarena and municipalities.

#### Definition of management problems

The planetary boundaries framework can support setting management objectives to steer economic activities such as agriculture and energy generation, to reduce pressures on the nitrogen and phosphorus cycles, water use, and land system in the river basin. For instance, poor management practices in agriculture are a main cause of the disturbed nitrogen and phosphorus cycles. These management practices include either an excess in the application or a deficit in the use of both nutrients. Excessive fertilizer use is pushed by the increasing demand for food, biofuels, and improved grass species to feed cattle in the river basin. A deficit on nitrogen and phosphorus is the consequence of extractive agriculture and livestock production (e.g., overgrazing), practices that remove nutrients from soil, plants, and animals. Poor agricultural management practices are also connected to pressures on water use and deforestation. However, the planetary boundaries framework provides little guidance on optimizing ecosystem management (e.g., fertilizer use) within the boundaries. In addition, it is far from straightforward to translate coarse-scale thresholds to management objectives for local-scale natural resource managers. In the context of ecosystem accounting, management objectives can be defined on the basis of targets related to ecosystem extent (e.g., forest cover), condition, biodiversity (provided a spatial, comprehensive biodiversity account has been developed), carbon, or potentially also services flow (United Nations et al. 2014b). Given that the accounts relate ecosystems and the economy, and that information can be expressed in both physical and monetary terms, accounting information can be used as input into economic optimization models. However, the accounts, if used in this way, implicitly assume a high degree of substitutability between different ecosystem assets (a weak sustainability interpretation).

Nevertheless, ecosystem accounting can be used to document extent, condition, and the capacity of ecosystems to supply ES over time, and as such, support monitoring. For example, ecosystem accounting specifies the growth in oil palm plantations in the Orinoco as an important driver for environmental change, increasing from 53,000 ha in 2004 to 174,000 ha in 2014 (Dane and Ministry of Agriculture 2016).

#### Establishment of a baseline for understanding and identification of alternatives for the Orinoco

**Fig. 1:**
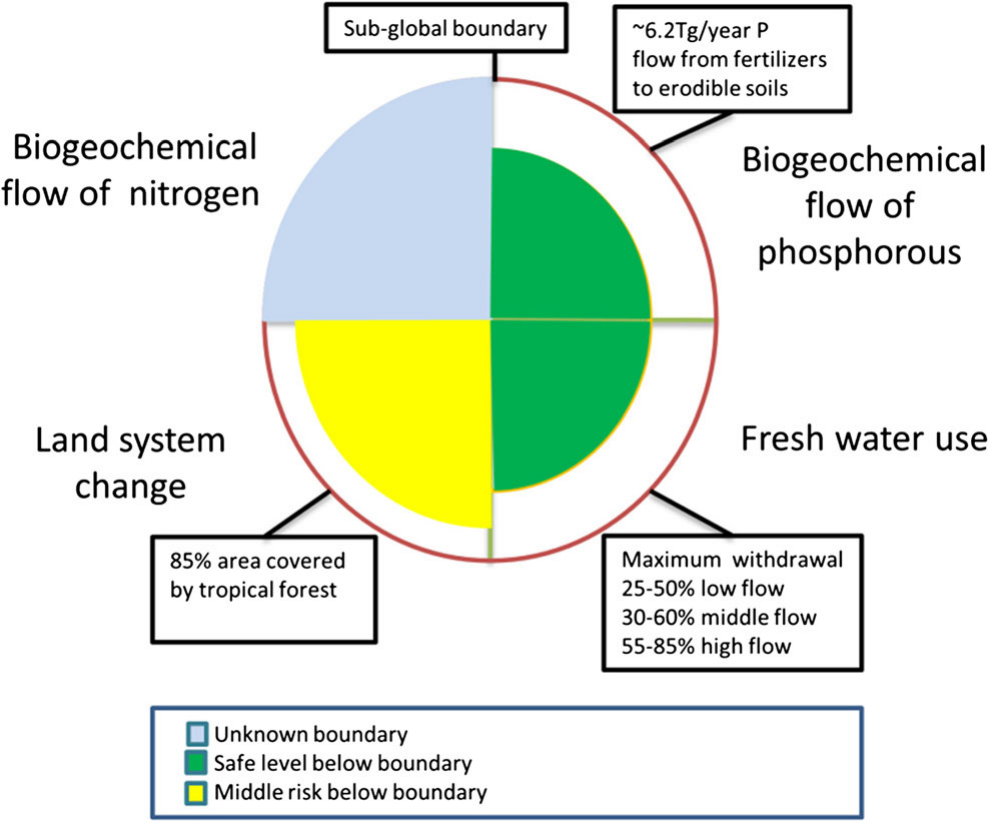
Boundary levels for earth system processes with sub-global dynamics. The size of the wedges show the position of the control variable for the Orinoco river basin

The planetary boundaries framework uses control variables to quantify the value of each boundary. Steffen et al. (2015) propose a set of control variables for sub-global processes based on expert assessment and current scientific knowledge. The level for four control variables of the planetary boundaries can help to explore the establishment of a baseline of understanding between the different stakeholders to evaluate alternative policy actions in the river basin (Fig. 1).

Two control variables were introduced by Steffen et al. (2015) to quantify the boundary associated to nitrogen and phosphorus cycles: (i) the industrial and biological fixation of nitrogen (mainly for agriculture uses) and (ii) the flow of total phosphorus from fertilizers applied on erodible soils. The first variable is estimated at a planetary level (62 Tg of nitrogen fixated per year (de Vries et al. 2013)) (Fig. 1). The second variable is estimated to have ~ 6.2 Tg of phosphorus per year as regional boundary to avoid eutrophication of freshwater systems, as the addition of phosphorus to river basins is almost entirely from fertilizers (Carpenter and Bennett 2011; Steffen et al. 2015). The values for both variables are not known for the Orinoco. However, modeled estimates show a nitrogen fixation around 0.3 Tg of nitrogen per year, and a flow of phosphorus of around 0.02 Tg of phosphorus per year in the year 2000, projected to reach 0.45 Tg of nitrogen per year, and 0.035 Tg of phosphorus per year by 2025 in the Orinoco basin (Camargo and Alonso 2006; van der Struijk and Kroeze 2010). To set the boundaries for land system change, Steffen et al. (2015) proposes as a control variable the area of forested land as a percentage. The boundary proposed by Steffen et al. (2015) is 85% of the remaining area of tropical forests (Fig. 1). Currently, 93% of the tropical forests in 2000 remained in 2015 in the river basin (Ideam 2011a, 2015a). Although the area covered by tropical forests is below the boundary, the risk of reaching the boundary in the coming years is high, as deforestation continues in the river basin (Ideam 2015a). For water use, Steffen et al. (2015) propose as the control variable the percentage of water withdrawal of monthly river flows (Gerten et al. 2013; Pastor et al. 2014). The water use boundary was estimated by Steffen et al. (2015) as 25% (25–55%) for low flow months, 30% (30–60%) for intermediate flow months, and 55% (55–85%) for high flow months. Current estimations for the main rivers of the river basin (e.g., Casanare, Arauca, and Meta) show water withdrawals of 50% for low flow months, 20% for intermediate flow months, and 50% for high flow months (Ideam 2011b, 2015b). Although water use is still below the basin boundary, the increasing demand for fresh water driven by increases on human activities (e.g., hydropower generation and irrigation) put a major pressure, especially in low flow months.

**Fig. 2:**
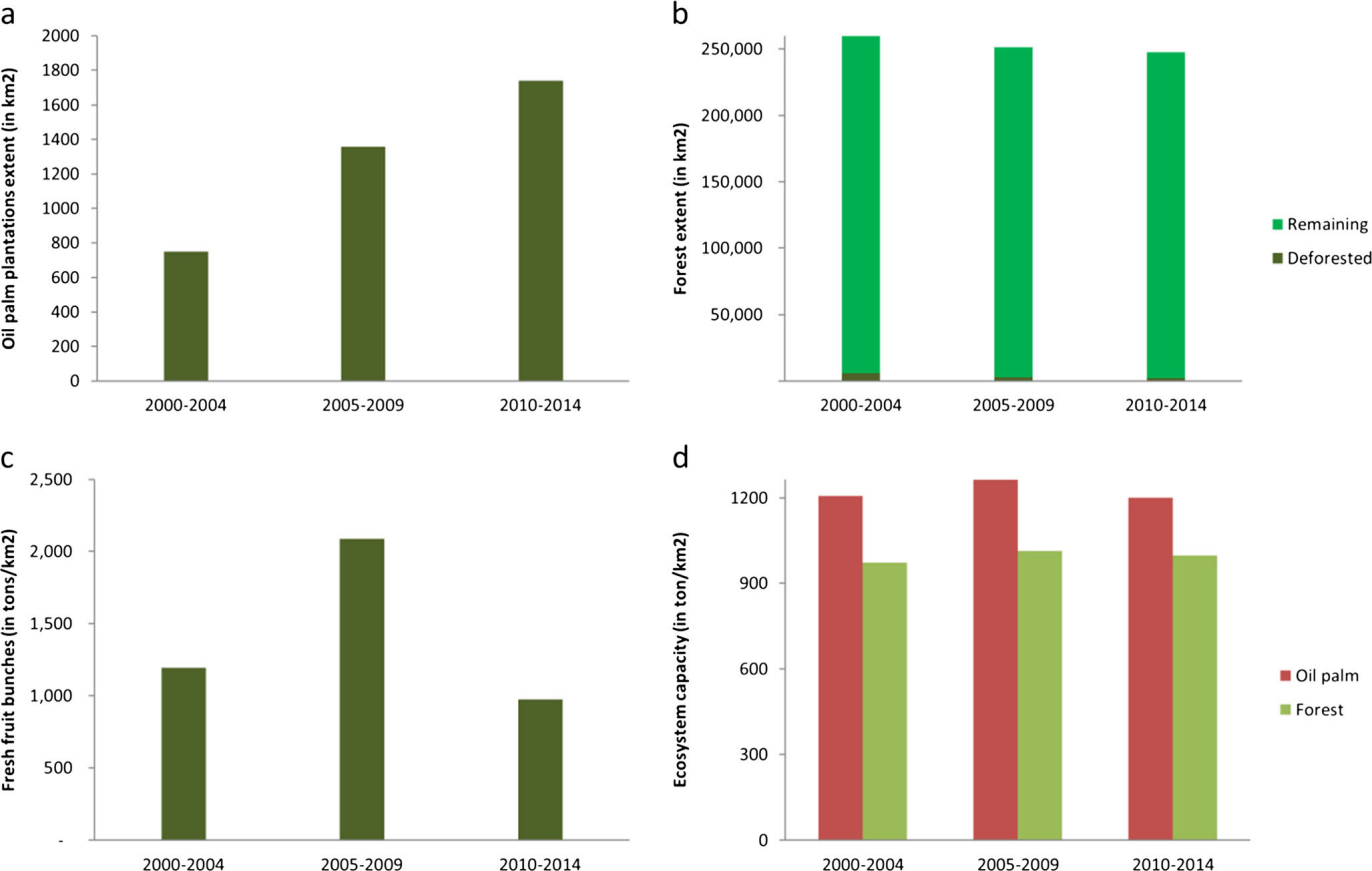
Time series between 2000 and 2014 for the Colombian Orinoco basin showing **a** changes in the extent of oil palm plantations (Fedepalma 2005; Fedepalma 2008; Fedepalma 2015) **b** changes in the extent of forest and the extent of deforested areas (Ideam 2011a, 2015a), **c** changes in the supply of oil palm fresh fruit bunches (Fedepalma 2005; Fedepalma 2008; Fedepalma 2015), and **d** changes in the capacity of oil palm plantations and forest to supply ecosystem services based on net primary productivity (NPP) (Vargas et al. 2017)

The biophysical information recorded in ecosystem accounting tables and accounts allows the assessment of changes in ecosystem’s extent, condition, and the future supply of ecosystem services. This information can be used to inform policy discussions, enabling the establishment of a baseline of understanding between different stakeholders to evaluate alternative policy actions in the river basin. For example, the ecosystem extent accounts for the Orinoco show the location of oil palm plantations and forest ecosystems, and the measurement of changes in extent over different accounting periods (Fig. 2). Information recorded in condition accounts allows the assessment of changes in ecosystem characteristics, such as changes in land cover by switching from forests to oil palm plantations. Trade-offs between the different ecosystem services supplied by alternative uses of ecosystems can be established, for example, changes in carbon sequestration, timber harvesting, erosion control, and flood regulation supplied by forests versus an increase in the supply of fresh fruit bunches from oil palm plantations (Vargas et al. 2017) (Fig. 2). This is an example that shows the effect of combining both frameworks in order to highlight how short-term, immediate resource gains are traded off against the long-term stability and complexity of the interacting processes that regulate the earth system.

### Implementation of actions or policies

Information from the boundary levels can be used to target specific policies in Colombia, including water regulation (Ministry of environment housing and land 2010), forest and land use (Ministry of environment et al. 2000; Ministry of environment and sustainable development 2013), and the nitrogen and phosphorus cycles (Ministry of environment and sustainable development 2013). Different actions can be implemented to reduce the current rates of deforestation, and regulate water use and the application of fertilizers containing nitrogen and phosphorus. Current policies related to the biogeochemical cycle of nitrogen and phosphorus are still weak at the national level. Information provided with ecosystem accounting can be used for decision and policy making including land use alternatives, alternative energy use, and long-term environmental trends awareness (Edens and Hein 2013; Obst and Vardon 2014; United Nations et al. 2014a). Trade-offs between the supply of ecosystem services (e.g., flood control in savannahs ecosystems versus rice production) and changes in ecosystem’s condition (e.g., nutrient depletion by poor agriculture practices) recorded in the different accounts can be used by governmental institutions such as autonomous regional environmental corporations (e.g., Corporinoquia, Cormacarena, Corpochivor) that regulate the use of natural resources in the Orinoco river basin.

### Monitoring effects

The control variables proposed by Steffen et al. (2015) such as phosphorus flows from fertilizers to erodible soils can be used to evaluate the impact of policy actions aiming to reduce the use of phosphorus in agriculture and eutrophication of lakes and rivers (Ministry of environment and sustainable development 2013; Ministry of environment housing and land 2010). Moreover, control variables such as area of forested land as a percentage of the original forest cover can be used to evaluate changes in the amount of tropical forest in the river basin, assessing the impact of forest conservation strategies. Ecosystem accounting provides consistent, spatially explicit, structured data over specific accounting periods useful to support monitoring strategies concerning ecosystem use and economic performance. Land use and land cover changes such as switching from forests to increase the extent of oil palm plantations can be monitored by crossing information recorded in extent and condition accounts every year. However, ecosystem accounting requires geo-referenced data and modeled outcomes which are not always available, especially in low-income countries. Ecosystem accounting provides biophysical information useful to measure the effectiveness of management options by looking at resource impacts and benefits during each accounting period.

## Discussion

### Planetary boundaries and ecosystem accounting

Implementing the planetary boundaries framework for achieving sustainability is challenged by the absence of defined boundaries for earth system processes for sub-global dynamics. This is relevant because surpassing local ecological thresholds can lead to local transitions in ecosystems, as well as because decisions concerning the governance and management of natural resources are mostly taken at sub-global level. Hence, translating global planetary boundaries to boundaries relevant at the national and sub-national levels is of utmost importance, and this has also been an active field of research (Cole et al. 2014; Dearing et al. 2014). Translating approaches either involves disaggregating global values by downscaling the control variables into (sub-)national targets or aggregation by determining indicators at the (sub-)national level (Häyhä et al. 2016). Our study shows that the aggregated approach applied by ecosystem accounting can support identifying thresholds, and monitoring progress towards maintaining ecological stability, in particular at the national or sub-national level.

However, contextual and structural differences between both approaches can either constrain or facilitate their potential integration. Contextual differences such as the purpose of analysis, methods, scale, and orientation are a major constraint to their integration. This is apparent, in particular, in the conceptualization of the social and ecological systems. The social system in the planetary boundaries framework is, in general, somewhat poorly conceptualized, focusing on the pressures resulting from economic activities on earth system processes. In this way, the accounting structure applied in ecosystem accounting complements the planetary boundaries framework by providing detailed economic information derived from economic transactions recorded in institutional units at the national level. The ecological system in the planetary boundaries framework focuses on key earth system processes. Biophysical information compiled in ecosystem accounting condition accounts can be relevant in assessing earth system processes with sub-global dynamics, by providing spatially explicit information concerning flows of nitrogen and phosphorus, evapotranspiration patterns, and water availability. The condition account, in particular, can be developed in such a way that it comprises information on pressures exerted on ecosystems, changes in state indicators, and potentially (although this has never been tested yet) a comparison between the current ecosystem condition with the condition at which ecological thresholds are likely to be exceeded (using a relative metric, see United Nations et al. 2017).

Furthermore, According to Häyhä et al. (2016), spatially heterogeneous interconnected processes (e.g., biogeochemical cycle of nitrogen and phosphorus) are only recently seen as global problems; however, they may not show up as national issues if only territorial approaches are applied at the national level. The planetary boundaries framework can be a powerful tool to translate spatially heterogeneous interconnected problems such as land system change and water use from global problems to national issues. Ecosystem accounting can be a powerful tool to support the planetary boundaries framework by incorporating global problems into information systems in support of national policies. Recent developments in earth observation systems including drones and satellite remote sensing (e.g., Landsat 8, Sentinel family missions) provide new data to populate condition and other accounts increasing the usefulness of ecosystem accounting also for monitoring ecosystem state in relation to planetary boundaries.

### Natural resource management in the Colombian Orinoco river basin

Given the rapid changes in land use in the area, management actions are needed to reduce human pressures in the biogeochemical cycle of nitrogen and phosphorus, land system change, and water use in the Orinoco river basin. Our study includes adaptive management criteria as described by Rist et al. (2013a) to assess the applicability of the planetary boundaries and ecosystem accounting frameworks for natural resource management in the Orinoco river basin. A current national policy CONPES (2014), has identified three million hectares in the Orinoco river basin as potential land to be converted from forests and savannahs to agriculture fields, without assessing the environmental responses following these changes. Potential impacts derived from implementing this policy include doubling the annual yield of nitrogen and phosphorus, reducing the total area covered by tropical forests, and reaching the maximum water withdrawal threshold in low flow months (Ideam 2011a, 2015a; van der Struijk and Kroeze 2010).

Turning planetary boundaries into management actions in the Orinoco river basin is challenged by difficulties in identifying stakeholders, uncertainties in defining the level of the boundaries and associated thresholds, and difficulties in developing monitoring strategies based on uncertain thresholds. Ecosystem accounting is an analysis-oriented framework that can provide useful information to overcome these challenges. Economic information compiled in institutional units can be used to identify relevant stakeholders in the river basin by identifying groups of economic activity such as the oil palm industry and farmers associations (e.g., FEDEPALMA and FEDEGAN), as well as, among others, profits and employment generated by these economic activities. Moreover, the accounting structure in ecosystem accounting allows aggregating source information to derive indicators, enabling the use of control variables such as phosphorus flow from fertilizers to erodible soils and nitrogen fixation as indicators to monitor changes in condition in aquatic and soil ecosystems in the river basin.

Condition accounts can be used to monitor changes in the river basin ecosystems caused by external pressures in planetary processes, and shifts in ecosystems quality aggregating biophysical information in condition indicators (e.g., evapotranspiration, water stress, drought, water quality, and biodiversity indicators). Setting limits on economic activities based on boundaries associated to nitrogen and phosphorus cycles, water use, and land system change, supported by information compiled in an ecosystem accounting structure, can be a promising approach for natural resource management at the level of river basin. However, in the case of the Orinoco basin, measurement of such flows is incomplete at the moment, and where measurements are available, there is no structured and regular reporting on these data. Ecosystem accounting in combination with the planetary boundary approach can assist in identifying key data gaps. Ecosystem accounting can also support developing assessment and communication approaches for such information (building on experiences with the national accounts of that in Colombia, as in most other countries, is reported on an annual basis following a specific set of guidelines including on data quality assurance).

## Conclusions

Achieving global sustainable development requires humanity to manage ecosystems in such a way that critical thresholds are avoided. Challenges occur both in defining these thresholds and in managing the trade-offs involved in resource management within the safe operating space. A solid monitoring system is required in order to assess how far the social-ecological system is removed from these thresholds and to guide policy actions in the environmental space. Our study postulates that ecosystem accounting can be used to support the translation of planetary boundaries into indicators that can be monitored at the national level, including boundaries for the nitrogen and phosphorus cycles, water use, and land system change. The concisely organized structure of ecosystem accounting accounts provides consistent information necessary to support decisions concerning environmental management and environmental policy and can facilitate the potential use of planetary boundaries at the national level. As we have shown with an application in the Orinoco river basin, shifting the traditional approach to governance and management towards a sustainable use of natural resources requires a combination of analytical and action-oriented frameworks to better inform decision makers. Although both the planetary boundaries and the ecosystem accounting frameworks pursue different purposes, supporting the achievement of sustainable development can be seen as a common ground between the two frameworks. Given strengths and weaknesses of both approaches, their combination is strongly recommended. Specifically, the planetary boundaries framework involves a stronger interpretation of sustainability compared to the ecosystem accounting framework and allows understanding environmental risks, whereas the ecosystem accounting offers a comprehensive monitoring framework as well as an opportunity to balance trade-offs within humanity’s safe operating space.
